# Supplementary material for: HSP70/DNAJ Family of Genes in the Brown Planthopper, Nilaparvata lugens: Diversity and Function
Source: Genes (Basel). 2021 Mar 10;12(3):394. doi: 10.3390/genes12030394 (PMC7999391; doi:10.3390/genes12030394)
Supplement: Supplementary file 1 [file genes-12-00394-s001.pdf]

Table S1： The 38 HSP70 homologs derived from 4 species and 70 DNAJ homologs derived from 5 species (homologs in *Nilaparvata lugens* not included).

**Family/ Subfamily**	**Name**	**Species**	**GenBank accession number**
HSP70	DmHSP70Aa	*Drosophila melanogaster*	NP_731651.1
	DmHSP70Ba	*Drosophila melanogaster*	NP_731716.1
	DmHSC70-1	*Drosophila melanogaster*	sp|P29843.1|
	DmHSC70-2	*Drosophila melanogaster*	sp|P11146.2|
	DmHSC70-3	*Drosophila melanogaster*	NP_727563.1
	DmHSC70-4	*Drosophila melanogaster*	sp|P11147.3|
	DmHSC70-5	*Drosophila melanogaster*	sp|P29845.2|
	DmHSP68	*Drosophila melanogaster*	NP_524474.1
	DmHSC70Cb	*Drosophila melanogaster*	CAB38172.2
	DmGRP170	*Drosophila melanogaster*	CG2918
	DmCG7182	*Drosophila melanogaster*	CG7182
	TcHSC70-1	*Tribolium castaneum*	EFA08256.1
	TcHSC70-2	*Tribolium castaneum*	XP_973521.1
	TcHSC70-3	*Tribolium castaneum*	XP_015838368.1
	TcHSC70-4	*Tribolium castaneum*	XP_966611.1
	TcHSC70	*Tribolium castaneum*	QCI56581.1
	TcHSP70A1	*Tribolium castaneum*	XP_974442.1
	TcHSP70Ab	*Tribolium castaneum*	EFA06541.1
	TcHSP68	*Tribolium castaneum*	EFA07510.1
	TcHSP68like	*Tribolium castaneum*	KYB26038.1
	HhHSC70-2	*Halyomorpha halys*	KAE8573711.1
	HhHSC70-4	*Halyomorpha halys*	XP_014281829.1
	HhHSC70-5	*Halyomorpha halys*	XP_014279735.1
	HhHSP70-4	*Halyomorpha halys*	XP_014280439.1
	HhHSP68	*Halyomorpha halys*	XP_014288075.1
	HhHSP68like	*Halyomorpha halys*	XP_014272950.1
	HhHSP70Ba	*Halyomorpha halys*	XP_014281580.1
	ZnHSC70-2	*Zootermopsis nevadensis*	XP_021939718.1
	ZnHSC70-3	*Zootermopsis nevadensis*	KDR23518.1
	ZnHSC70-4	*Zootermopsis nevadensis*	KDR23254.1
	ZnHSC70-5	*Zootermopsis nevadensis*	XP_021939160.1
	ZnHSP70A1	*Zootermopsis nevadensis*	XP_021938753.1
	ZnHSP70-4L	*Zootermopsis nevadensis*	KDR19282.1
DNAJA	AaDNAJA1	*Aedes aegypti*	XP_021704647.1
	AgDNAJA1	*Aphis gossypii*	XP_027841039.1
	AmDNAJA1	*Apis mellifera*	XP_016767139.1
	TcDNAJA1	*Tribolium castaneum*	XP_971446.1
	TcDNAJA2	*Tribolium castaneum*	EFA03800.1
DNAJB	AaDNAJB6	*Aedes aegypti*	XP_021707244.1
	AaDNAJB12	*Aedes aegypti*	XP_001663382.1

	AaDNAJB13	*Aedes aegypti*	XP_001655507.2
	AaDNAJB14	*Aedes aegypti*	XP_001656767.1
	AgDNAJB6	*Aphis gossypii*	XP_027846547.1
	AgDNAJB13	*Aphis gossypii*	XP_027851793.1
	AgDNAJB14	*Aphis gossypii*	XP_027851994.1
	AmDNAJB6	*Apis mellifera*	XP_026297956.1
	AmDNAJB12	*Apis mellifera*	XP_006561461.1
	AmDNAJB13	*Apis mellifera*	XP_001123348.1
	TcDNAJB6	*Tribolium castaneum*	XP_008197911.1
	TcDNAJB11	*Tribolium castaneum*	EFA03492.2
	TcDNAJB12	*Tribolium castaneum*	EFA01075.2
	TcDNAJB13	*Tribolium castaneum*	XP_969979.1
DANAJC	AaDNAJC3	*Aedes aegypti*	XP_021705034.1
	AaDNAJC5	*Aedes aegypti*	XP_001653560.1
	AaDNAJC8	*Aedes aegypti*	XP_001650318.1
	AaDNAJC11	*Aedes aegypti*	XP_021706038.1
	AaDNAJC16	*Aedes aegypti*	XP_001655444.2
	AaDNAJC17	*Aedes aegypti*	XP_001647945.1
	AaDNAJC22	*Aedes aegypti*	XP_001661613.2
	AaDNAJC30	*Aedes aegypti*	XP_001661210.1
	AgDNAJC1	*Aphis gossypii*	XP_027843483.1
	AgDNAJC3	*Aphis gossypii*	XP_027852438.1
	AgDNAJC8	*Aphis gossypii*	XP_027848680.1
	AgDNAJC9	*Aphis gossypii*	XP_027840965.1
	AgDNAJC11	*Aphis gossypii*	XP_027845038.1
	AgDNAJC13	*Aphis gossypii*	XP_027836804.1
	AgDNAJC16	*Aphis gossypii*	XP_027841337.1
	AgDNAJC17	*Aphis gossypii*	XP_027845018.1
	AgDNAJC22	*Aphis gossypii*	XP_027843274.1
	AgDNAJC30	*Aphis gossypii*	XP_027852604.1
	AmDNAJC1	*Apis mellifera*	XP_624533.1
	AmDNAJC3	*Apis mellifera*	XP_006569244.2
	AmDNAJC5	*Apis mellifera*	XP_026301299.1
	AmDNAJC7	*Apis mellifera*	XP_026298080.1
	AmDNAJC8	*Apis mellifera*	XP_006558850.1
	AmDNAJC9	*Apis mellifera*	XP_393383.3
	AmDNAJC11	*Apis mellifera*	XP_393479.3
	AmDNAJC13	*Apis mellifera*	XP_016767742.2
	AmDNAJC16	*Apis mellifera*	XP_395584.4
	AmDNAJC17	*Apis mellifera*	XP_625225.1
	AmDNAJC21	*Apis mellifera*	XP_016769063.1
	AmDNAJC22	*Apis mellifera*	XP_006568607.1
	AmDNAJC28	*Apis mellifera*	XP_026295176.1
	BmDNAJC3	*Bombyx mori*	NP_001296552.1

BmDNAJC10	*Bombyx mori*	XP_012546160.2
BmDNAJC13	*Bombyx mori*	XP_012549735.1
BmDNAJC16	*Bombyx mori*	NP_001296558.1
BmDNAJC28	*Bombyx mori*	NP_001296541.1
TcDNAJC1	*Tribolium castaneum*	XP_974769.2
TcDNAJC3	*Tribolium castaneum*	EFA11836.2
TcDNAJC5	*Tribolium castaneum*	XP_015836237.1
TcDNAJC7	*Tribolium castaneum*	EFA04042.1
TcDNAJC8	*Tribolium castaneum*	EEZ98101.1
TcDNAJC10	*Tribolium castaneum*	XP_008190269.1
TcDNAJC11	*Tribolium castaneum*	XP_008193614.1
TcDNAJC13	*Tribolium castaneum*	EFA06092.2
TcDNAJC16	*Tribolium castaneum*	EFA10042.1
TcDNAJC17	*Tribolium castaneum*	EFA00119.1
TcDNAJC21	*Tribolium castaneum*	EFA06925.1
TcDNAJC22	*Tribolium castaneum*	EFA00002.1
TcDNAJC28	*Tribolium castaneum*	EFA08155.1
TcDNAJC30	*Tribolium castaneum*	EFA05522.1
TcSEC63	*Tribolium castaneum*	EFA04351.2

Table S2: The main primers used in this study.

**Primer name**	**Sequence (5'-3')***	**Length**	**Purpose**
qNlHSC70-F	ACTTGTCTGGGACGCTGCTA	113bp	qRT-PCR for *NlHSP70s*
qNlHSP70-R	TGGAGAACGGCTCTTTGC		
qNlHSC70-2-F	ATGATGTCGTGCTCGTTGG	110bp	
qNlHSC70-2-R	CGCCTCGTCTGGGTTGAT		
qNlHSC70-3-F	TGTTGGTTGGCGGTAGCA	166bp	
qNlHSC70-3-R	ACAATGGCGTCGGTCTTC		
qNlHSC70-4-F	CTTCGATTTGGGTGGTGG	169bp	
qNlHSC70-4-R	CAGGTCCTTCTTGTGCTTCC		
qNlHSC70-5-F	CAGGCGATACGAAGATGC	184bp	
qNlHSC70-5-R	CTGCGAACCGAGATAGGA		
qNlHSC70-6-F	GAGCCTGGATGAGCACGAC	155bp	
qNlHSC70-6-R	CGCATAGGGCAAGAATAGC		
qNlHSP68-F	TCGGTGGGTCAACTCGTA	150bp	
qNlHSP68-R	AAGCTCCATTGTCTCCTGTC		
qNlHSP70A-F	GCAGTCATCACCGTTCCAG	186bp	
qNlHSP70A-R	ACCACCTCCCAGGTCAAAA		
qNlHSP70B-F	GAATCTTTCAATCAACCCTG	146bp	
qNlHSP70B-R	CTCCTGCTGTCTCAATACCTAA		
qNlDNAJA1-F	CGGGCGACATCATCATAG	132bp	qRT-PCR for *NlDNAJs*
qNlDNAJA1-R	GGGTTCGGATCACCTTTT		
qNlDNAJA2-F	GACTGCTGTGATACCTCCCG	106bp	
qNlDNAJA2-R	CTCGTCGTCATCGTCGTTC		

qNlDNAJA3-F	CATCATCGCATACTCGCATAA	169bp
qNlDNAJA3-R	CCAGGCGTGTCATCTTCC	
qNlDNAJB5-F	TTCCGCTCCCAGTCGTT	157bp
qNlDNAJB5-R	CGTCAGGCTGCATCACC	
qNlDNAJB6-F	TCTATTACTTTCTGTGCCCTAT	115bp
qNlDNAJB6-R	TTCGGTTGATGCCTGTTT	
qNlDNAJB9-F	CATCCCTCTTTCCATCCA	178bp
qNlDNAJB9-R	TGCTGTGCTCGTGTTCTC	
qNlDNAJB11-F	GACAAGAACCGAGACAACCC	190bp
qNlDNAJB11-R	CATCGCCAAAGTGAAATCC	
qNlDNAJB12-F	AGGGTCAGTCACAGGCACA	120bp
qNlDNAJB12-R	CGAGAAACCTCCACCAAAA	
qNlDNAJB13-F	CGGAGACCCAGTCAGAACC	181bp
qNlDNAJB13-R	TTTGATGGCACCCAAGTAA	
qNlDNAJC1-F	CTCCAAACGGCAACAACG	111bp
qNlDNAJC1-R	TCCTCCTCCACCTCCATC	
qNlDNAJC2-F	CGACAATGTGCCCTCCAATA	191bp
qNlDNAJC2-R	AATACTCCCTCCACGACTCAA	
qNlDNAJC3-F	GGTCCAGCTTTGCCAGTG	207bp
qNlDNAJC3-R	CCCTCCTTCGCCCTCTT	
qNlDNAJC5-F	ATCCCAACAATCCAGAAGC	177bp
qNlDNAJC5-R	ACCAGCCAGATGTGACGAG	
qNlDNAJC7-F	AATCGCTCCAGATCATCGT	173bp
qNlDNAJC7-R	GCTTGGCATTTGTGACTTT	
qNlDNAJC8-F	GCACGCTGTCTATGTGATG	125bp
qNlDNAJC8-R	TCTCCTCCTCTTCTATTTCTTG	
qNlDNAJC9-F	GCAGGAAACGGTGGGAGA	194bp
qNlDNAJC9-R	GCAGTATTTGGCGGTGAGA	
qNlDNAJC10-F	CCACCAACCTAAGAACCATT	168bp
qNlDNAJC10-R	GGACTGAGGCGTCAAACA	
qNlDNAJC11-F	GACGGTGACTCCGCTGTTT	122bp
qNlDNAJC11-R	CTGCCATTCGTTTCTTGTTGA	
qNlDNAJC12-F	GACCTCTACGCCATCCTCG	117bp
qNlDNAJC12-R	GGCCTCCTTGTTTCCTTCA	
qNlDNAJC13-F	TCAAGCGGGAGTTGTCTG	119bp
qNlDNAJC13-R	GTTGTCCTCGGCAATGTG	
qNlDNAJC14-F	ATGTGACCGATGACAAGAATG	179bp
qNlDNAJC14-R	TGCCGTGGTTGCTGAGTAG	
qNlDNAJC16-F	TCCACAAGATGTCCGTCAG	105bp
qNlDNAJC16-R	AGGCGAAGCACCAGTCC	
qNlDNAJC17-F	ATGGCGGCTATGATGAAG	166bp
qNlDNAJC17-R	GGATTCTCAGCAAACCCT	
qNlDNAJC19-F	GCAGGGATAGGATTGGTGGT	173bp
qNlDNAJC19-R	CGTCTTGTCATCTTCGGCTC	

qNlDNAJC21-F	GATGACGGTGAAGACTACGA	105bp	
qNlDNAJC21-R	TTCTGGCATTGAGGAGGA		
qNlDNAJC22-F	GAGCCAATCAAACAGAAATCA	164bp	
qNlDNAJC22-R	CCTTCGCCTCTTCACCTTAC		
qNlDNAJC23-F	ACATTGTCCTCATTTCCCTG	125bp	
qNlDNAJC23-R	GCACCTCCTCGTGTTCCA		
qNlDNAJC28-F	GTTTGACCACCACAATCCC	165bp	
qNlDNAJC28-R	CTGCCCAGCCTACTCCTTT		
qNlDNAJC30-F	GCAACATCAATCGCAAGAA	119bp	
qNlDNAJC30-R	CCCATACTGAGGCACAGAG		
qNlDNAJC31-F	CATAACAAAGACCCGGAAGC	176bp	
qNlDNAJC31-R	GCCGCACCTTGAACAGC		
qNlDNAJC32-F	AAACAATACCATCCCGACCAT	194bp	
qNlDNAJC32-R	GCCGCACCTTGAACAGC		
qNl18S-F	CGCTACTACCGATTGAA	132bp	qRT-PCR for *Nl18s*
qNl18S-R	GGAAACCTTGTTACGACTT		
NlHSC70-F	AGGAAACAGGACGACACC	354 bp	PCR for NlHSP70s
NlHSP70-R	TTGAAGTAGGCTGGAACG		
NlHSC70-2-F	CGGTCCTATTCCCTATTCA	648bp	
NlHSC70-2-R	GCTCATCCAGGCTCGTTAT		
NlHSC70-3-F	AAGGCACTCCCAGACATTC	484bp	
NlHSC70-3-R	GGCATTCAGCCAAGTTAGA		
NlHSC70-4-F	CACCTACTCGGACAATCAGC	283bp	
NlHSC70-4-R	CTCGGCGTCGTTCACCAT		
NlHSC70-5-F	CACGACACGGAGACCAAGA	237bp	
NlHSC70-5-R	GCTGCTACTGCTTCCGCTAC		
NlHSC70-6-F	ATCCAGGTATTTGAGGGTGA	442bp	
NlHSC70-6-R	ATGTGGCTTGTGGGTTGTT		
NlHSP68-F	CATAATGAATCAGATGGAAAG	218bp	
NlHSP68-R	GATGAATTTTGACTAAAACGAC		
NlHSP70A-F	ACAACACCGAAGAGGAACA	670bp	
NlHSP70A-R	TGCCTAATCTGGGCTACA		
NlHSP70B-F	ACCAGAACCGACTGACGC	235bp	
NlHSP70B-R	TCTTCCAGCCATTTGATTT		
NlDNAJA1-F	TCTCCAGCTTCTTCGGATTT	274bp	PCR for NlDNAJs
NlDNAJA1-R	CGCATAACATTGGGACATTC		
NlDNAJA2-F	TCTGACCTGCCATCCTGAC	221bp	
NlDNAJA2-R	ATTCTGCTGCTCGTTCCCT		
NlDNAJA3-F	ACTTCACCGCTGACGATT	575bp	
NlDNAJA3-R	CGATGATGTTCCCTCTGG		
NlDNAJB5-F	GCATTCCAGTTGTTCGCTAC	514bp	
NlDNAJB5-R	TGGTCGTCATCCGTTTCC		
NlDNAJB6-F	ATTCGTGTCGGTCCTGTAA	409bp	

NlDNAJB6-R	TCTCGTCCGTCAAGGTGTT	
NlDNAJB9-F	CAAGCCAAAGAAGTGAGTGT	370bp
NlDNAJB9-R	TACGATGATCTGGAGCGATT	
NlDNAJB11-F	GAACTGGTAGGGTTTGTGAG	704bp
NlDNAJB11-R	AGAACAGGGTCATCGTAGTC	
NlDNAJB12-F	GCTTCCCTCCTGGATTGTTG	422bp
NlDNAJB12-R	CGTTGTCAGTTGGTCTTTCG	
NlDNAJB13-F	GAACGACGCCGCCAGTA	238bp
NlDNAJB13-R	CGAAGCACCAGTCCGAGTA	
NlDNAJC1-F	AAGACGGGAAGCCAGTTTA	126bp
NlDNAJC1-R	GCTGCAAGGTAGGGTGATC	
NlDNAJC2-F	AGGCATCAATCTTCTGACA	435bp
NlDNAJC2-R	GTTTGGTGGGCATCTGGT	
NlDNAJC3-F	TCTGTTGTGGGAGGTAGTGG	277bp
NlDNAJC3-R	CATACGCCTTCTGGTTGG	
NlDNAJC5-F	AGAAACGAGCCAACTACGA	294bp
NlDNAJC5-R	TGCGGAACTTGCTGACG	
NlDNAJC7-F	TGTCAACGCTTACTTCCTCG	293bp
NlDNAJC7-R	TCACCGCCTCGCTCATAC	
NlDNAJC8-F	AGTATGGGAGGACTCCTTACTATGA	120bp
NlDNAJC8-R	CTATTTCGCTTCGTTTCAGGTTA	
NlDNAJC9-F	ACCAGTGGAGGCGACAAC	307bp
NlDNAJC9-R	TAGCACGTACTTCGTAGATT	
NlDNAJC10-F	GCCGACTACTGGCGATGA	443bp
NlDNAJC10-R	TGGCGGAATGGTGTATGTT	
NlDNAJC11-F	TGCTCCACCGCTCACTTA	412bp
NlDNAJC11-R	GCGCACCATGTTGTCTTT	
NlDNAJC12-F	CAGGCACCCATTATTTCT	445bp
NlDNAJC12-R	ATTGCTGGTGTTGAGGAG	
NlDNAJC13-F	TGGTGCAGCTACTTGTACTTC	258bp
NlDNAJC13-R	TTCTGTTTGATTGGCTCTTG	
NlDNAJC14-F	AGAAGTTCGTAAAGCCTATC	470bp
NlDNAJC14-R	TCTTGGGTGACTCGTTGT	
NlDNAJC16-F	ATCCAAAGGGTGAAAGGT	640bp
NlDNAJC16-R	ACGAAGCTCGGGTAGTGC	
NlDNAJC17-F	CTATGATAAGTACGGCGAGGAG	318bp
NlDNAJC17-R	AACGACTGGGAGCGGAAA	
NlDNAJC19-F	AAAGCGTGACTCTGTTCTG	364bp
NlDNAJC19-R	CTCCTCTACTTGCCCATCT	
NlDNAJC21-F	GTTCAGCCAATACGAAGATG	594bp
NlDNAJC21-R	TACTCAAACAATGCTCCAAA	
NlDNAJC22-F	AGCAGGCACTGAAGGAGG	414bp

NlDNAJC22-R	CTTTCATTCCGTTACAGGTTTT			
NlDNAJC23-F	CTCTGAGGACAGGTGGAAA	444bp		
NlDNAJC23-R	ACATAGGGATTGTGGTGGT			
NlDNAJC28-F	AAATCACGAGCCCTTTACG	459bp		
NlDNAJC28-R	CCCACTGGAATCACTACCG			
NlDNAJC30-F	ATGTACGACGAAGCAATAAA	418bp		
NlDNAJC30-R	ACTTGAACTGGAAGGGACT			
NlDNAJC31-F	GCAAACAGGCATCAACCG	316bp		
NlDNAJC31-R	GACCCGCCAAAGAACTCC			
NlDNAJC32-F	TTCTATGAGGTGCTTGGTG	290bp		
NlDNAJC32-R	TTTGGATGGAAAGAGGGAT			
NlHSC70-RNAi-F	T7-AGGAAACAGGACGACACC	354 bp	NlHSP70s synthesis	dsRNA
NlHSP70-RNAi-R	T7-TTGAAGTAGGCTGGAACG			
NlHSC70-2-RNAi-F	T7-CGGTCCTATTCCCTATTCA	648bp		
NlHSC70-2-RNAi-R	T7-GCTCATCCAGGCTCGTTAT			
NlHSC70-3-RNAi-F	T7-AAGGCACTCCCAGACATTC	484bp		
NlHSC70-3-RNAi-R	T7-GGCATTCAGCCAAGTTAGA			
NlHSC70-4-RNAi-F	T7-CACCTACTCGGACAATCAGC	283bp		
NlHSC70-4-RNAi-R	T7-CTCGGCGTCGTTCACCAT			
NlHSC70-5-RNAi-F	T7-CACGACACGGAGACCAAGA	237bp		
NlHSC70-5-RNAi-R	T7-GCTGCTACTGCTTCCGCTAC			
NlHSC70-6-RNAi-F	T7-ATCCAGGTATTTGAGGGTGA	442bp		
NlHSC70-6-RNAi-R	T7-ATGTGGCTTGTGGGTTGTT			
NlHSP68-RNAi-F	T7-CATAATGAATCAGATGGAAAG	218bp		
NlHSP68-RNAi-R	T7-GATGAATTTTGACTAAAACGAC			
NlHSP70A-RNAi-F	T7-ACAACACCGAAGAGGAACA	670bp		
NlHSP70A-RNAi-R	T7-TGCCTAATCTGGGCTACA			

NlHSP70B-RNAi-F	T7-ACCAGAACCGACTGACGC	235bp	
NlHSP70B-RNAi-R	T7-TCTTCCAGCCATTTGATTT		
NlDNAJA1-RNAi-F	T7-TCTCCAGCTTCTTCGGATTT	274bp	NlDNAJs dsRNA synthesis
NlDNAJA1-RNAi-R	T7-CGCATAACATTGGGACATTC		
NlDNAJA2-RNAi-F	T7-TCTGACCTGCCATCCTGAC	221bp	
NlDNAJA2-RNAi-R	T7-ATTCTGCTGCTCGTTCCCT		
NlDNAJA3-RNAi-F	T7-ACTTCACCGCTGACGATT	575bp	
NlDNAJA3-RNAi-R	T7-CGATGATGTTCCCTCTGG		
NlDNAJB5-RNAi-F	T7-GCATTCCAGTTGTTCGCTAC	514bp	
NlDNAJB5-RNAi-R	T7-TGGTCGTCATCCGTTTCC		
NlDNAJB6-RNAi-F	T7-ATTCGTGTCGGTCCTGTAA	409bp	
NlDNAJB6-RNAi-R	T7-TCTCGTCCGTCAAGGTGTT		
NlDNAJB9-RNAi-F	T7-CAAGCCAAAGAAGTGAGTGT	370bp	
NlDNAJB9-RNAi-R	T7-TACGATGATCTGGAGCGATT		
NlDNAJB11-RNAi-F	T7-GAACTGGTAGGGTTTGTGAG	704bp	
NlDNAJB11-RNAi-R	T7-AGAACAGGGTCATCGTAGTC		
NlDNAJB12-RNAi-F	T7-GCTTCCCTCCTGGATTGTTG	422bp	
NlDNAJB12-RNAi-R	T7-CGTTGTCAGTTGGTCTTTCG		
NlDNAJB13-RNAi-F	T7-GAACGACGCCGCCAGTA	238bp	
NlDNAJB13-RNAi-R	T7-CGAAGCACCAGTCCGAGTA		
NlDNAJC1-RNAi-F	T7-AAGACGGGAAGCCAGTTTA	126bp	
NlDNAJC1-RNAi-R	T7-GCTGCAAGGTAGGGTGATC		

NlDNAJC2-RNAi-F	T7-AGGCATCAATCTTCTGACA	435bp
NlDNAJC2-RNAi-R	T7-GTTTGGTGGGCATCTGGT	
NlDNAJC3-RNAi-F	T7-TCTGTTGTGGGAGGTAGTGG	277bp
NlDNAJC3-RNAi-R	T7-CATACGCCTTCTGGTTGG	
NlDNAJC5-RNAi-F	T7-AGAAACGAGCCAACTACGA	294bp
NlDNAJC5-RNAi-R	T7-TGCGGAACTTGCTGACG	
NlDNAJC7-RNAi-F	T7-TGTCAACGCTTACTTCCTCG	293bp
NlDNAJC7-RNAi-R	T7-TCACCGCCTCGCTCATAC	
NlDNAJC8-RNAi-F	T7-AGTATGGGAGGACTCCTTACTATGA	120bp
NlDNAJC8-RNAi-R	T7-CTATTTCGCTTCGTTTCAGGTTA	
NlDNAJC9-RNAi-F	T7-ACCAGTGGAGGCGACAAC	307bp
NlDNAJC9-RNAi-R	T7-TAGCACGTACTTCGTAGATT	
NlDNAJC10-RNAi-F	T7-GCCGACTACTGGCGATGA	443bp
NlDNAJC10-RNAi-R	T7-TGGCGGAATGGTGTATGTT	
NlDNAJC11-RNAi-F	T7-TGCTCCACCGCTCACTTA	412bp
NlDNAJC11-RNAi-R	T7-GCGCACCATGTTGTCTTT	
NlDNAJC12-RNAi-F	T7-CAGGCACCCATTATTTCT	445bp
NlDNAJC12-RNAi-R	T7-ATTGCTGGTGTTGAGGAG	
NlDNAJC13-RNAi-F	T7-TGGTGCAGCTACTTGTACTTC	258bp
NlDNAJC13-RNAi-R	T7-TTCTGTTTGATTGGCTCTTG	
NlDNAJC14-RNAi-F	T7-AGAAGTTCGTAAAGCCTATC	470bp

NlDNAJC14-RNAi-R	T7-TCTTGGGTGACTCGTTGT	
NlDNAJC16-RNAi-F	T7-ATCCAAAGGGTGAAAGGT	640bp
NlDNAJC16-RNAi-R	T7-ACGAAGCTCGGGTAGTGC	
NlDNAJC17-RNAi-F	T7-CTATGATAAGTACGGCGAGGAG	318bp
NlDNAJC17-RNAi-R	T7-AACGACTGGGAGCGGAAA	
NlDNAJC19-RNAi-F	T7-AAAGCGTGACTCTGTTCTG	364bp
NlDNAJC19-RNAi-R	T7-CTCCTCTACTTGCCCATCT	
NlDNAJC21-RNAi-F	T7-GTTCAGCCAATACGAAGATG	594bp
NlDNAJC21-RNAi-R	T7-TACTCAAACAATGCTCCAAA	
NlDNAJC22-RNAi-F	T7-AGCAGGCACTGAAGGAGG	414bp
NlDNAJC22-RNAi-R	T7-CTTTCATTCCGTTACAGGTTTT	
NlDNAJC23-RNAi-F	T7-CTCTGAGGACAGGTGGAAA	444bp
NlDNAJC23-RNAi-R	T7-ACATAGGGATTGTGGTGGT	
NlDNAJC28-RNAi-F	T7-AAATCACGAGCCCTTTACG	459bp
NlDNAJC28-RNAi-R	T7-CCCACTGGAATCACTACCG	
NlDNAJC30-RNAi-F	T7-ATGTACGACGAAGCAATAAA	418bp
NlDNAJC30-RNAi-R	T7-ACTTGAACTGGAAGGGACT	
NlDNAJC31-RNAi-F	T7-GCAAACAGGCATCAACCG	316bp
NlDNAJC31-RNAi-R	T7-GACCCGCCAAAGAACTCC	
NlDNAJC32-RNAi-F	T7-TTCTATGAGGTGCTTGGTG	290bp
NlDNAJC32-RNAi-R	T7-TTTGGATGGAAAGAGGGAT	

*T7, 5´-TAATACGACTCACTATAGGGAGA-3´
